# *Panicle Apical Abortion 3* Controls Panicle Development and Seed Size in Rice

**DOI:** 10.1186/s12284-021-00509-5

**Published:** 2021-07-15

**Authors:** Fayu Yang, Mao Xiong, Mingjiang Huang, Zhongcheng Li, Ziyi Wang, Honghui Zhu, Rui Chen, Lu Lu, Qinglan Cheng, Yan Wang, Jun Tang, Hui Zhuang, Yunfeng Li

**Affiliations:** Rice Research Institute, Key Laboratory of Application and Safety Control of Genetically Modified Crops, Academy of Agricultural Sciences, Southwest University, Chongqing, 400715 China

**Keywords:** Rice, Panicle apical abortion, Programmed cell death, H^+^-ATPase

## Abstract

**Background:**

In rice, panicle apical abortion is a common phenomenon that usually results in a decreased number of branches and grains per panicle, and consequently a reduced grain yield. A better understanding of the molecular mechanism of panicle abortion is thus critical for maintaining and increasing rice production.

**Results:**

We reported a new rice mutant *panicle apical abortion 3* (*paa3*), which exhibited severe abortion of spikelet development on the upper part of the branches as well as decreased grain size over the whole panicle. Using mapping-based clone, the *PAA3* was characterized as the *LOC_ Os04g56160* gene, encoding an H^+^-ATPase*.* The *PAA3* was expressed highly in the stem and panicle, and its protein was localized in the plasma membrane. Our data further showed that *PAA3* played an important role in maintaining normal panicle development by participating in the removal of reactive oxygen species (ROS) in rice.

**Conclusions:**

Our studies suggested that *PAA3* might function to remove ROS, the accumulation of which leads to programmed cell death, and ultimately panicle apical abortion and decreased seed size in the *paa3* panicle.

**Supplementary Information:**

The online version contains supplementary material available at 10.1186/s12284-021-00509-5.

## Background

Rice (*Oryza sativa*) is one of the most important food crops in the world and the major staple food for more than half the world’s population (Takeda and Matsuoka, 2008). Rice yield is determined by three main component traits: number of panicles, number of grains per panicle, and grain weight (Xing and Zhang, 2010). Therefore, the panicle plays a key role in contributing directly to yield, and the achievement of optimal panicle structure, size, and shape is one of the goals in high-yield breeding (Sakamoto and Matsuoka, 2004).

In the development of the inflorescence, the transition from shoot meristems to axillary meristems (AMs) determines the complexity of the inflorescence architecture (Huijser and Schmid, 2011). The rice panicle has a four-order inflorescence structure, comprising the main axis, primary branches (PBs), secondary branches (SBs), lateral spikelet (LS), and terminal spikelet (TS), and all of which are originated from the AMs. The past few years have seen the identification of a number of regulatory genes involved in the determination of panicle architecture, including lymphocyte transmembrane adaptor 1 (*LAX1)*, *LAX2*, and *MONOCULM 1*. These genes participate in the initiation, formation, and maintenance of AMs in the rice panicle and are regulators of panicle architecture. They all encode transcription factor and the loss-of-function mutants display greatly reduced numbers of branches and spikelets (Komatsu et al., 2003; Li et al., 2003; Tabuchi et al., 2011). The transition from branch meristem to spikelet meristem is also considered a key process related to panicle architecture. *RETICULOCALBIN 1 (RCN1)* and *RCN2* maintain the branch meristem and control its fate in rice, and their overexpression leads to the delayed transition from branch meristem to spikelet meristem, and then to more branches and spikelets (Nakagawa et al., 2002). *ABBERANT PANICLE ORGANIZATION 2* (*APO2)/RFL* can be activated by the DNA-binding one zinc finger DOF-domain transcription activator SHORT PANICLE 3 (SP3), and interacts with APO1 to delay the transition from branch meristem to spikelet meristem. In the mutants *apo1*, *apo2* and *sp3*, the numbers of branches and spikelets are all significantly reduced (Rao et al., 2008; Ikeda-Kawakatsu et al., 2012). In addition, *TAW1* encodes a nuclear protein that participates in determining the fate of the branch meristem. In the gain-of-function mutant *taw1-d*, the branch meristem activity is increased and the differentiation of the spikelet meristem is delayed, which promotes the formation of more SBs and spikelets (Yoshida et al., 2013). Plant hormones such as cytokinin also play crucial roles in panicle architecture. *Grain number 1a* (*Gn1a*), a major quantitative trait locus for grain number per panicle, encodes cytokinin oxidase which is responsible for the degradation of cytokinin. In the natural allelic variant with low expression of *Gn1a*, the accumulation of cytokinin leads to greatly increase branch number, spikelet number, and grain yield (Ashikari et al., 2005). Multiple genes such as *DROUGHT AND SALT TOLERANCE* (*DST*), *VIN3-LIKE 2* (*VIL2*) and *LARGER PANICLE* (*LP*) (Li et al., 2011; Li et al., 2013; Yang et al., 2019) participate in panicle development by regulating the expression of *Gn1a*. The microRNA156- IDEAL PLANT ARCHITECTURE 1-DENSE AND ERECT PANICLE 1 (MicroRNA156-IPA1-DEP1) pathway probably regulates panicle development through cytokinin, in which *IPA1* can directly activate *DEP1* expression by interacting with its promoter, and then the high expression of *DEP1* represses *Gn1a* (Huang et al., 2009; Lu et al., 2013).

After the panicle architecture is established, normal spikelet growth is very important for the final yield. Panicle abortion occurs at either the top or basal parts of the panicle. Spikelet growth often stops at the apex of the panicle and/or branches under disadvantageous conditions (malnutrition, extreme temperatures, shading, and water stress) and genetic alteration, which is usually termed “panicle apical abortion” (Kobayasi et al., 2001; Kato et al., 2008; Bai et al., 2015). To date, only a few studies have set out to characterize the molecular mechanism of panicle abortion. The *TOTOU1* gene was the first cloned pleiotropy gene associated with panicle degeneration, through its encoding of cyclic adenosine monophosphate receptor protein inhibitors. *TOTOU1* mutation leads to panicle apical abortion and other defects including tiller reduction, leaf tip degeneration, and dwarfing (Bai et al., 2015). *SQUAMOSA PROMOTERBINDING PROTEIN-LIKE 6* (*SPL6*) functions as a transcriptional repressor of *INOSITOL-REQUIRING ENZYME (IRE1)*, and acts as an essential survival factor for the suppression of persistent or intense stress in the endoplasmic reticulum, leading ultimately to cell death in rice. The *spl6–1* mutant displays hyperactivation of *IRE1*, leading to cell death in spikelets in the panicle apex (Wang et al., 2018a, b). ALUMINUM-ACTIVATED MALATE TRANSPORTER 7 (OsALMT7) is a malate transporter that functions in the development of panicle apical portions, and its mutation also results in reduced malate and cell death, particularly at the apical portion of the panicle (Heng et al., 2018). The *paa1019* mutant is specifically defective in panicle development. *PAA1019* encodes OsCIPK31, a calcineurin B-like-interacting protein kinase that affects the development of panicle apical spikelets (Peng et al., 2018). The *degenerated panicle and partial sterility 1* (*dps1*) mutant also show panicle apical degeneration and reduced fertility in middle spikelets. In addition, the amounts of cuticular wax and cuticle are reduced significantly in *dps1* anthers, and the accumulation of reactive oxygen species (ROS), lower antioxidant activity, and increased programmed cell death (PCD) have all been observed (Zafar et al., 2019).

In this study, we report a novel rice mutant *panicle apical abortion 3* (*paa3*), which exhibits the degeneration of spikelets at the tops of panicles during the late stage of panicle development. The results of gene cloning and complementation tests indicate that *PAA3* is *LOC_Os04g56160*, encoding an H^+^-ATPase. Our data further suggest that *PAA3* might function to remove peroxides, and the accumulation of ROS leads to PCD and ultimately panicle apical abortion in the *paa3* mutant.

## Results

### The *paa3* Mutant Displayed a Semi-Dwarf Phenotype and Severe Panicle Apical Abortion

**Fig. 1:**
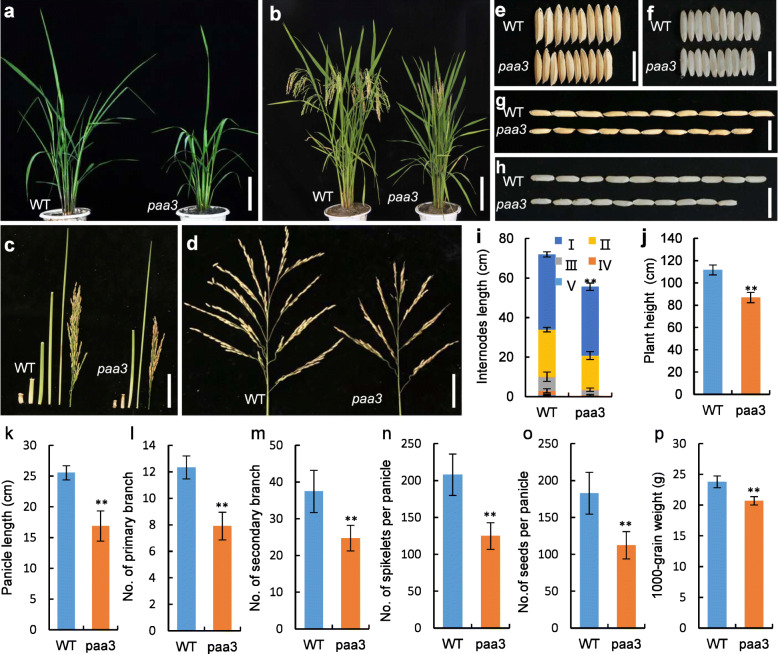
Phenotypic characterization of the rice (*panicle apical abortion3*) *paa3* mutant. **a**: Morphology of wild-type (WT) and *paa3* plant seedling stage. **b**: Morphology of WT and *paa3* plant at maturity. **c**: Internodes and panicle of WT and *paa3*. **d**: Panicle branch architectures of WT and *paa3*. **e**: Comparison of grain morphology width between WT and *paa3*. **f** Comparison of brown rice morphology width between WT and *paa3*. **g**: Comparison of grain morphology length between WT and *paa3*. **h**: Comparison of brown rice morphology length between WT and *paa3*. **i**: Panicle length. **j**: Plant height. **k**: Panicle height. **l**: Number of primary branch. **m**: Number of secondary branch. **n**: Total number of spikelets. **o**: Number of seeds per panicle. **p**: 1000 grain weight. Bars: (**a**, **b**)10 cm; (**c**, **d**) 5 cm; (**e**–**h**) 1 cm. ** *p* < 0.01 (Student’s t-test)

**Fig. 2:**
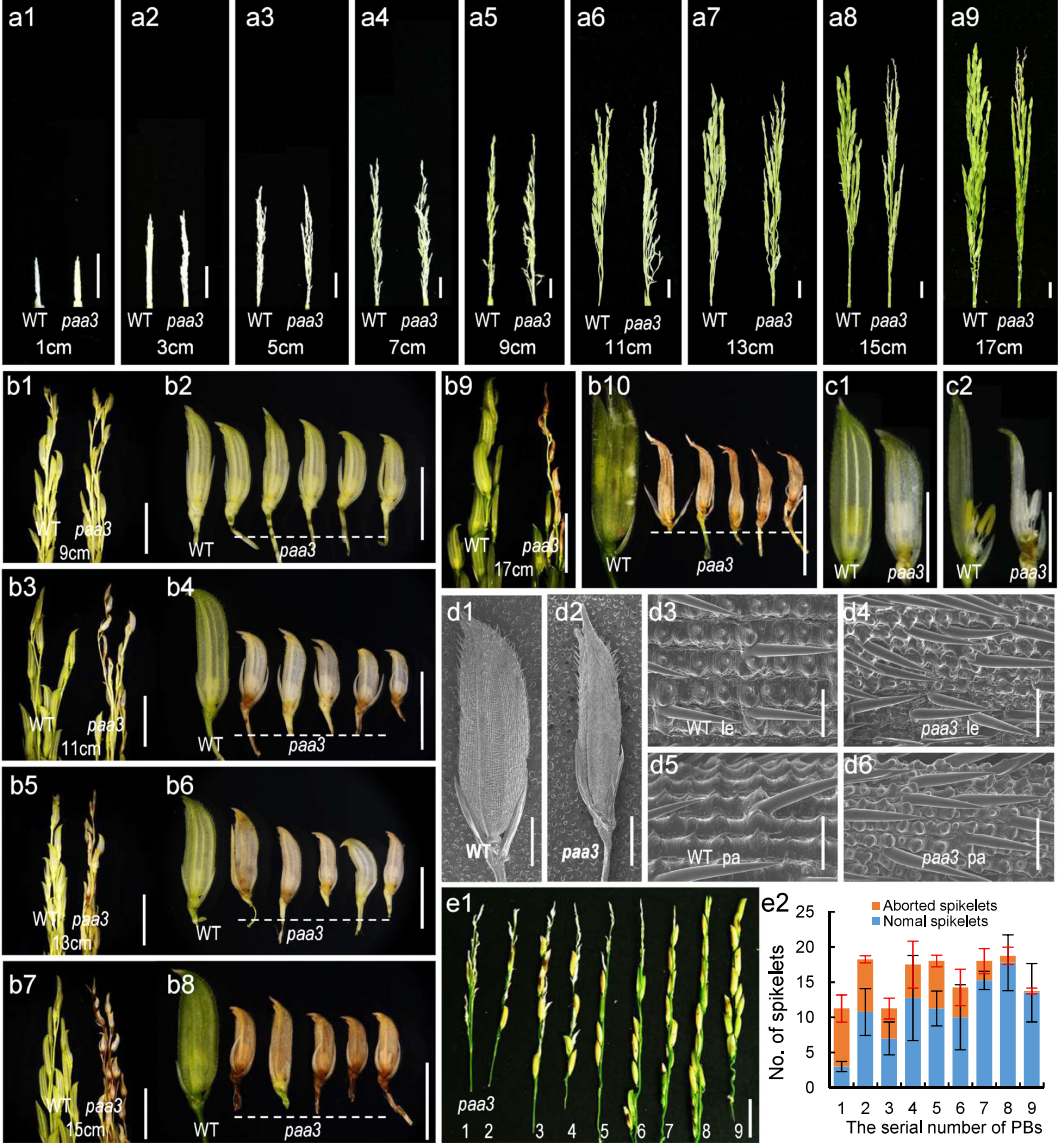
Characteristics of abortion spikelets. **a**: Representative images of WT (left) and *paa3* (right) developing panicles, showing different stages as indicated by panicle length:1 cm (**a**1), 3 cm (**a**2), 5 cm (**a**3), 7 cm (**a**4), 9 cm (**a**5), 11 cm (**a**6), 13 cm (**a**7), 15 cm (**a**8) and 17 cm(**a**9). **b**: The apical developing panicles, showing different stages as indicated by panicle length: 9 cm (**b**1, **b**2),11 cm (**b**3, **b**4),13 cm (**b**5, **b**6),15 cm (**b**7, **b**8),17 cm (**b**9, **b**10). **c**: Top spikelet in *paa3* mutants and WT; Total spikelet(**c**1), Spikelet without lemma(**c**2). (panicle length = 11 cm): **d**: Scanning electron microscopy observation of top spikelet in *paa3* mutant and WT: total spikelet of WT(**d**1), total spikelet of *paa3*(**d**2), WT lemma (**d**3), *paa3* lemma (**d**4), WT palea (**d**5). *paa3* palea (**d**6). **e**: Analysis of the aborted panicle in *paa3*: The image of the primary branch of *paa3* panicle; Arabic numerals (1–9) denote the serial number of PBs(e1), Analysis of degenerated spikelets in each primary branch of *paa3* in **e**1(**e**2). Bars: (**a**1–**a**9, e1)1cm; (**b**1–**b**10 and **c**1–**c**2)5mm; (**d**1–**d**2) 2 mm; (**d**3–**d**4) 120 μm

The *paa3* mutant exhibited decreased plant height and grain defects (Fig. 1). The dwarf phenotype in the *paa3* mutant was first observed at the tillering stage (Fig. 1a), and the semi-dwarf phenotype lasted until maturity stage (Fig. 1b). Detailed analyses show that the lengths of both the internodes (from 1 to 4) and the panicle in the *paa3* mutant were all significantly reduced compared to the wild type (WT), and internode 5 was not elongated in the *paa3* mutant but was elongated in the WT (Fig. 1c, i). Both the grain number and size in the *paa3* mutant were also affected (Fig. 1e–h). The numbers of PBs and SBs were all sharply reduced compared with the WT (Fig. 1l, m), which resulted in a significant reduction in the spikelet number per panicle (including the aborted spikelet in *paa3* mutant) (Fig. 1n). The number of grains in the *paa3* panicles was also significantly decreased compared to the WT (Fig. 1o). In addition, both grain length and grain width, as well as 1000-grain weight in the *paa3* mutant were all significantly reduced compared to those in the WT (Fig. 1e–h, p). In particular, unlike the WT plants in which all the spikelets developed normally, obvious panicle apical abortion was found in the *paa3* panicle, in which the spikelets in the apical part of the panicle showed termination of development and were unable to seed (Fig. 1d). To determine when the abortion occurred, we investigated a series of mutant panicles at nine stages according to panicle length (~ 1, ~ 3, ~ 5, ~ 7, ~ 9, ~ 11, ~ 13, ~ 15, and ~ 17 cm). The *paa3* spikelets at the top of the panicles began to show developmental delay at 11 cm, and the abortion phenotype became increasingly obvious from ~ 13 to ~ 17 cm (Fig. 2a1–a9). We further observed in detail the spikelets at the top of the *paa3* panicles from ~ 9 to ~ 17 cm (Fig. 2b1–b10). At the ~ 9 cm panicles, there were no obvious differences between the WT and the *paa3* spikelets (Fig. 2b1, b2). However, some of the spikelets at the top of the ~ 11 and ~ 13 cm panicles displayed white hulls and smaller size in *paa3* mutants, whereas the WT had green hulls and were larger in size (Fig. 2b3–b6). In about ~ 15 cm *paa3* panicles, the spikelet organs at the top of the panicles began to browning (Fig. 2b7, b8), and in the ~ 17 cm *paa3* panicles, the spikelets were completely dry (Fig. 2b9, b10). According to these results, we speculated that the spikelets located at the top of the *paa3* panicles stopped growing at ~ 9 to 11 cm, after which cell death occurred gradually. We also observed apical spikelets with ~ 9 cm panicles in WT and *paa3* mutant. And found that the stamen exhibited a hydrated phenotype (Fig. 2c1-c2). We further used scanning electron microscopy to observe the hull development in the ~ 11 cm panicles and found the size of the epidemic cells in both the lemma and palea of *paa3* were smaller than those in the WT (Fig. 2d1–d6). We conducted statistics on the abortion rate of panicles of wild type and mutants, and found that the abortion rate of mutants could reach 18.25% (Table S1). In addition, we observed the degree of spikelet abortion among the PBs for *paa3* by statistical analysis of 15 *paa3* panicles from 15 individual plants at the mature stage. The degree of spikelet abortion increased gradually from the lower to the upper PBs, and the upper spikelets were degenerated at a higher rate than the lower ones (Fig. 2e1, e2).

### Map-Based Cloning of the *PAA3* Gene

**Fig. 3:**
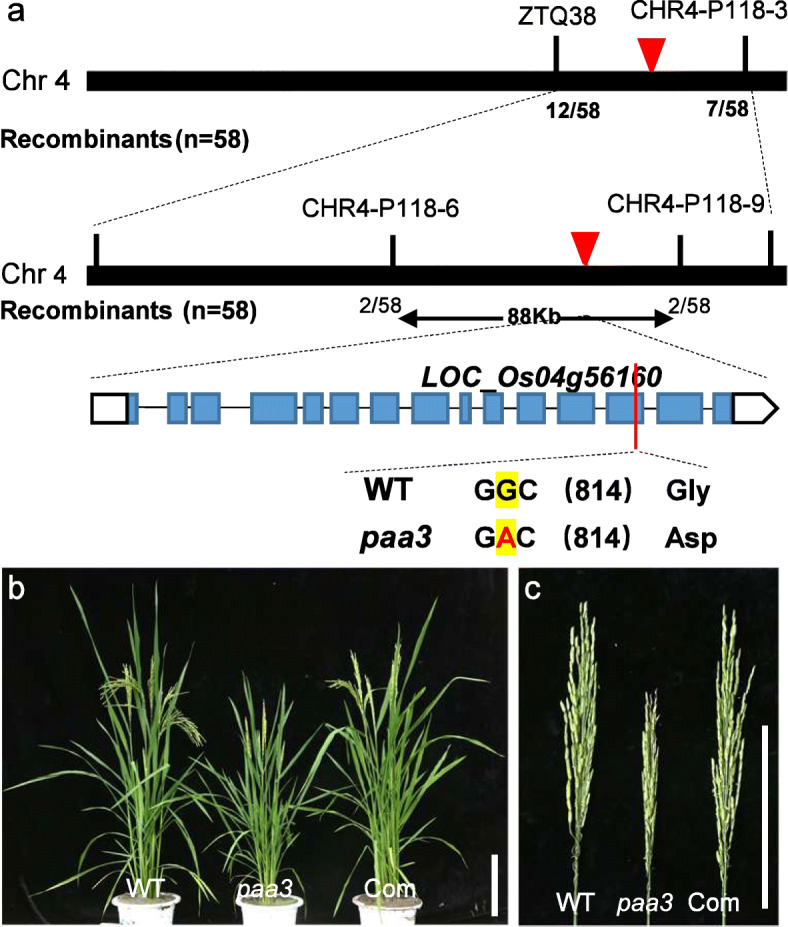
Molecular identification of *PAA3* and genetic complementation test. **a**: Fine mapping of the *PAA3* gene. **b**, **c**: Genetic complementation of *paa3*. **b**: Image of whole plants. **c**: Image of panicles. Bars (**b**, **c**) 15 cm

The *paa3* mutant was crossed with the sterile line 56 s to obtain the F1 generation, and the F1 generation was self-crossed to obtain the F2 population. All F1 plants exhibited a normal phenotype, while trait segregation occurred in the F2 population. The number of plants in the F2 population was 196, of which 138 were normal, 58 displayed *paa3* mutational phenotype, for a segregate ratio of 3.379:1, which was in accordance with the standard 3:1 of the chi-square test. Indicating that the mutant trait was controlled by a single recessive gene. In the F_2_ progeny, 58 individuals exhibiting a mutant phenotype were used as the mapping population. Using bulked segregant analysis, the *PAA3* gene was mapped to the long arm of chromosome 4 within an approximately 88 Kb region between the simple-sequence repeat (SSR) markers CHR4-P118–6 and CHR4-P118–9. In this region, a single-nucleotide substitution from G to A (Gly to Asp) was identified in the 13th exon of *LOC_Os04g56160* (Fig. 3a). The complementary expression vector containing the *LOC_Os04g56160* coding sequence (6406 bp), the 3251 bp upstream sequence from the start codon, and the 1068 bp downstream sequence from the stop codon was then transformed into the *paa3* mutants. In total, 28 transgenic plants were obtained, of which 15 showed rescued of the mutated phenotypes (Fig. 3b, c). We used two pairs of primers for amplification to detect the 15 transformants (comF1-GUSR1 for exogenous vector; comF2-comR2 for endogenic sites) (Fig. S1a). The sequencing results showed that the comF1-GUSR1 fragment was homozygous WT genotype and the comF2-comR2 fragment was homozygous mutation genotype, indicating that the exogenous complementary plasmid had been transformed successfully with the *paa3* mutant (Fig. S1b). Therefore, these results together indicated that *LOC_Os04g56160* was the *PAA3* gene.

### Expression Pattern Analysis and Subcellular Location of the *PAA3*

**Fig. 4:**
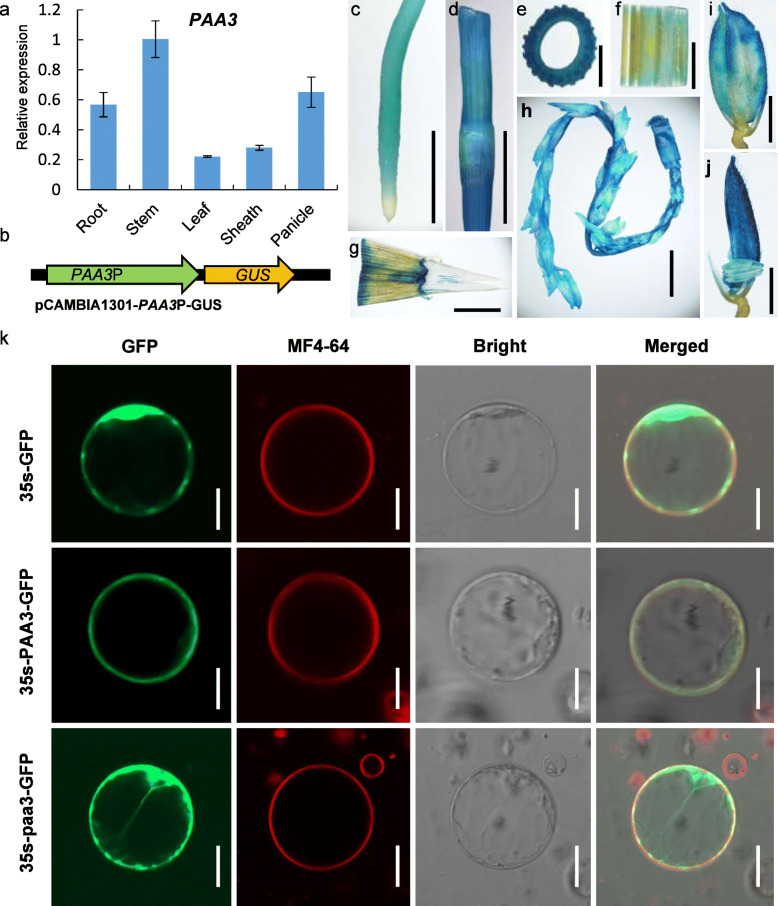
Expression pattern analysis of the *PAA3* gene and subcellular localization of PAA3 protein*.* **a**: qPCR analysis of rice *paa3* from different tissues of WT normalized to actin. **b**: Structure of *PAA3*P-GUS. **c**–**j**: Tissue-specific expression of the *GUS* gene driven by the rice *paa3* promoter. Root (**c**) Stem (**d**, **e**) Leaf blade (**f**) Sheath (**g**) Panicle (**h**), Spikelet (**i**) Spikelet without lemma (**j**). **k**: Analysis of the subcellular localization of the PAA3 protein. 35 s-GFP indicates the expression of GFP protein without PAA3 in rice protoplasts as the negative control. 35 s-PAA3-GFP indicates the plasma membrane localization of the PAA3 protein in rice protoplasts given by the expression of PAA3 fused with GFP. 35 s-paa3-GFP indicates the cytoplasm localization of the paa3 protein in rice protoplasts given by the expression of paa3 fused with GFP. The paa3 protein is the mutant protein. Green is GFP signal. Red is the plasma membrane signal, which was labelled by MF4–64. Bright is the bright light. Merged indicates the confused with the Green signal, the Red signal, and the bright light. Bars: (**c**–**h**) 10 mm; (**I** and **j**) 1 mm; (**k**) 50 μm

**Fig. 5:**
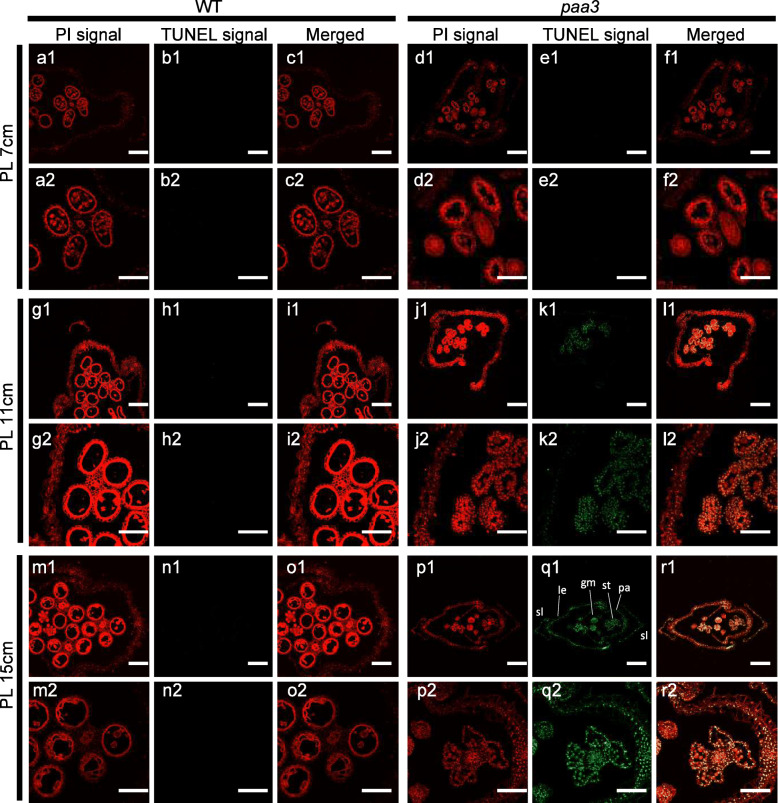
Detection of DNA fragmentation by the TUNEL assay. Green fluorescence indicates TUNEL-positive signals for DNA fragmentation, red fluorescence indicates staining of spikelets and with propidium iodide (PI), and yellow fluorescence results from overlay of green signals of TUNEL and red signals of PI staining. **a**1–**c**1: Top spikelets of WT in panicle length at 7 cm. **a**2–**c**2 enlargement of region (**a**1–**c**1). **d**1–**f**1: Top spikelets of *paa3* in panicle length at 7 cm. **d**2–**f**2 enlargement of region of (**d**1–**f**1). **g**1–**i**1: Top spikelets of WT in panicle length at 11 cm. **g**2–**i**2 enlargement of region (**g**1–**i**1). **j**1–**l**1 Top spikelets of *paa3* in panicle length at 11 cm. **j**2–**l**2 enlargement of region of (**j**1–**l**1). **m**1–**o**1: Top spikelets of WT in panicle length at 15 cm. **m**2–**o**2, enlargement of region (**m**1–**o**1). **p**1–**r**1: Top spikelets of *paa3* in panicle length at 15 cm. **p**2–**q**2 enlargement of region of (**p**1–**q**1). Bars: (**a**1–**r**1) 50 μm; (**a**2–**r**2) 10 μm. st: stamens; gy: gynoecium; sl: sterile lemma; le: lemma; pa: palea

To explore the spatiotemporal expression of *PAA3* in rice, we firstly applied quantitative PCR (qPCR) to examine *PAA3* expression in the WT plants. Expression of *PAA3* was detected in all rice organs analyzed, with relatively higher expression in the stems, roots, and panicles and lower expression in leaves and sheath (Fig. 4a). Next, we generated stable transgenic rice plants expressing the β-glucuronidase (GUS) reporter gene driven by a 3138 bp promoter sequence of *PAA3* (Fig. 4b)*.* Strong GUS staining was detected in the roots, stems, young panicles, and spikelets (Fig. 4c–e, h), while faint staining was observed in the leaves and sheathes (Fig. 5f, g), similar to the qPCR results. Notably, a strong GUS signal was detected in the hulls of the spikelets and stems (Fig. 4i–j).

In this study, to clarify the subcellular location of PAA3, we fused it with the green fluorescent protein (GFP) reporter gene and expressed the PAA3:GFP fusion protein in rice protoplasts. In cells expressing 35 s::PAA3:GFP protein, the GFP signal was observed mainly in the plasma membrane, and it was indeed colocalized with MF4–64 on the plasma membrane in the protoplasts (Fig. 4k). In the control group, 35 s promoter-driven GFP signals could be seen in the whole protoplast (Fig. 4k). In *Nicotiana benthamiana* leaves, the GFP fluorescence of 35 s::PAA3:GFP was also localized to the plasma membrane as labelled by MF4–64 (Fig. S2). These results suggested that PAA3 protein was localized to the plasma membrane.

In order to explore whether the change of single nucleotide affected the subcellular location of mutated PAA3 protein, we also fused it with the GFP reporter gene and expressed the paa3:GFP fusion protein in rice protoplasts (35 s::paa3:GFP Fig. 4k). The GFP signal of 35 s::paa3:GFP was located in the whole protoplasts highly consistent with the signal of 35 s::GFP. These results suggest that mutated paa3 protein lost the location of plasma membrane because of its the single nucleotide substitution from G to A.

### PCD Occurs in the Spikelets of *paa3* Panicles

As mentioned earlier, we found that the *paa3* spikelets at the top of the panicles began to show developmental delay at 11 cm (Fig. 2). Trypan blue staining was used to detect the level of cell death at ~ 13 cm in the WT and *paa3* spikelets at the top of the panicles, and significantly deeper staining was observed in the *paa3* spikelets than in the WT (Fig. S3). Therefore, these results indicated that the panicle apical abortion related to cell death in spikelet development during late *paa3* panicles development. To examine further if the panicle abortion phenotype was related to the PCD, we performed a terminal deoxynucleotidyl transferase-mediated dUTP nick-end labelling (TUNEL) assay, which showed nuclear DNA fragmentation at the single-cell level. In the ~ 7 cm panicles, no obvious TUNEL signal was detected in either the WT or the *paa3* spikelet (Fig. 5a1–f1, a2–f2). In the ~ 11 cm panicles, however, strong TUNEL signals were observed in the anthers of the *paa3* spikelets, and parts of the hull cells in the *paa3* spikelets also showed clear TUNEL signals (Fig. 5j1–l1, j2–l2), whereas no TUNEL signal was seen in the WT spikelets (Fig. 5 g1-i1, i2-i2). In the ~ 15 cm panicles, there was still no TUNEL signal in the WT spikelets (Fig. 5 m1–o1, m2–o2), but the whole of the spikelet (including hulls, stamens, gynoecium and sterile lemma) in the *paa3* mutant showed very strong TUNEL signals (Fig. 5p1–r1, p2–r2). These results suggest that DNA fragmentation and cell death started to occur in the *paa3* spikelets between the stages of ~ 7- ~ 11 cm, and reached a limitation at the stage of ~ 15 cm, consistent with the results of phenotypic analysis.

### Overaccumulation of ROS Induces PCD in Panicle Apical Spikelets

**Fig. 6:**
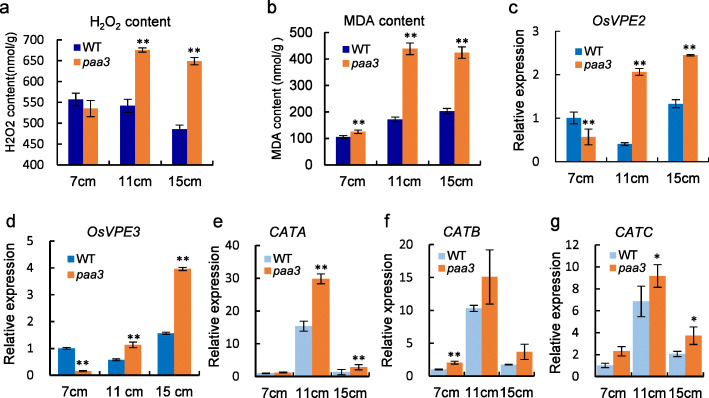
Cell death-related Events are induced in *paa3.* **a**: Measurement of H_2_O_2_ content in panicles of the 7, 11, and 15 cm stages in WT and *paa3*. **b**: Measurement of MDA content in panicles of the 7, 11, and 15 cm stages in WT and *paa3*, showing overaccumulation of ROS during late development stages in *paa3*. **c**: Expression level of *OsVPE2* in WT and *paa3* panicles at the 7, 11, and 15 cm stages. **d**: Expression levels of *OsVPE2* and *OsVPE3* in WT and *paa3* panicles at the 7, 11, and 15 cm stages, which showed PCD from the stage of 11 cm. **e**–**g**: Relative expression of *CATA*, *CATB*, and *CATC* isozymes. (rice *ACTIN* was used as the internal control). * indicates *p* < 0.05; ** indicates *p* < 0.01 (Student’s t-test)

ROS act as an important trigger of PCD, and excessive accumulation of hydrogen peroxide (H_2_O_2_) can trigger cell death (Mittler, 2017). We used DAB staining to qualitatively detect the content of H_2_O_2_. In the *paa3* panicles, DAB staining had revealed higher levels of ROS accumulation than that in the WT (Fig. S4). Here, we further measured H_2_O_2_ content in panicles of WT and *paa3*, and found an H_2_O_2_ blast at the ~ 11 cm stage in the *paa3* panicle (Fig. 6a). Malondialdehyde (MDA) accumulation is considered to be an indicator of lipid peroxidation and cell death (Chen and Murata, 2002). We therefore measured MDA content and found that the MDA levels were raised significantly in the 11 and 15 cm *paa3* panicles compared to the WT (Fig. 6b).

Next, some of the genes related to ROS or PCD were investigated. Vacuolar processing enzymes (VPEs) are involved in PCD in Arabidopsis (Kuroyanagi et al., 2005). There are only four VPE homologs (*OsVPE1, OsVPE2, OsVPE3, OsVPE4*) in rice, of which *OsVPE2* and *OsVPE3* play crucial roles in H_2_O_2_-induced PCD (Deng et al., 2011). We therefore measured the expression of both *OsVPE2* and *OsVPE3* in the WT and the *paa3* panicle at the ~ 7, ~ 11, and ~ 15 cm stages*.* The expression of *OsVPE2* in *paa3* was similar to that of the WT at the ~ 7 cm stage, but was increased significantly at the ~ 11 and ~ 15 cm stages compared to those of the WT (Fig. 6c). The expression of *OsVPE3* in the *paa3* panicles was lower than that in the WT panicles at the ~ 7 cm stage but was higher than that at the ~ 11 and ~ 15 cm stages (Fig. 6d). Catalase (CAT) is the key peroxidase in the biological defensive system, playing a role in converting excessive H_2_O_2_ into oxygen. *OsCATA*, *OsCATB*, and *OsCATC* encode CAT isozymes in rice (Zhang et al., 2016). Our results show that all three genes were expressed at higher levels in *paa3* than those in the WT panicles (Fig. 6e–g).

**Fig. 7:**
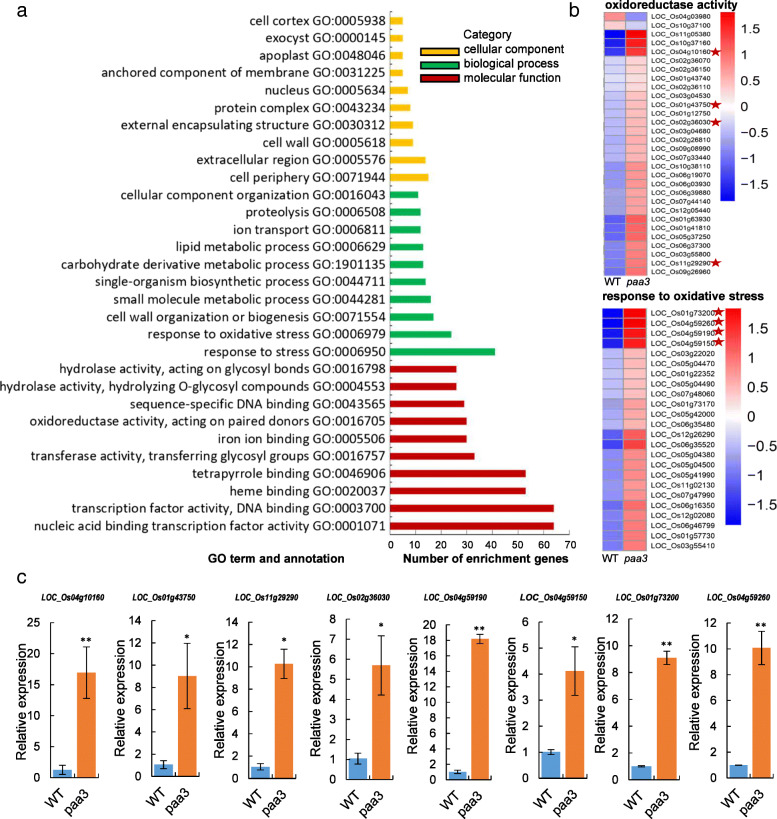
Transcriptome analysis (GO enrichment term). **a**: Comparison of GO enrichment analysis. The top 10 significantly enriched GO terms for cellular component (CC), biological process (BP), and molecular function (MF) in *paa3* mutant relative to normal panicle development in WT plants are shown in yellow, green, and red, respectively. **b**: Heat map based on FPKM values showing expression pattern of randomly selected genes involved in oxidoreductase activity (from FM) and response to oxidative stress (from BP). (Red and blue indicate the relative expression of one gene in WT and *paa3* mutant, respectively. The darker the color, the greater the difference) The yellow star represents the gene that we have picked out for qPCR verification. (The results have been shown in Fig. c) **c**: qPCR analysis of several genes related to the GO terms “oxidoreductase activity” and “response to oxidation.” * *p* < 0.05; ** *p* < 0.01 (Student’s t-test)

To provide further clarification of the mechanisms underlying ROS accumulation in the *paa3* panicle, we conducted transcriptome analysis. A total of 1075 differentially expressed genes (DEGs) were characterized, including 991 upregulated and 84 downregulated genes. The number of upregulated DEGs was 11.79 times the number of downregulated DEGs (Fig. S5). Next, Gene Ontology (GO) analysis showed that DEGs related to the oxidation response (GO: 0006979) and oxidoreductase activity (GO: 0016705) were enriched significantly, most of which were upregulated in the *paa3* mutant (Fig. 7a, b). Further qPCR was used to verify the expression of some of these DEGs including *LOC_Os04g10160*, *LOC_Os01g43750*, *LOC_Os11g29290*, and *LOC_Os02g36030* in “oxidoreductase activity”; and *LOC_Os04g59190*, *LOC_Os04g59150*, *LOC_Os01g73200*, and *LOC_Os04g59260* in response to “oxidative stress”. Compared to the WT, expression of *LOC_Os04g10160* and *LOC_Os04g59190* was upregulated nearly 20-fold in the *paa3* mutant, and expression of the other genes was upregulated more than 4-fold (Fig. 7c). These results suggested that *PAA3* played an important role in removal of ROS in rice.

## Discussion

In this study, we characterized a novel panicle development mutant *paa3*, which exhibited serious spikelet degeneration at the apical portion of panicle. Along with a reduced number of branches and 1000-grain weight, panicle apical portion resulted in a severe reduction of grain yield in the *paa3* mutant. During rice growth and development, panicle development is crucial for grain yield in rice. Panicle apical portion is a common reason for the low seed-setting rate in rice (Heng et al., 2018). A number of environmental, physiological, and genetic factors can cause panicle apical portion and reduction in grain yield including climatic conditions (Yao et al., 2000), hormonal imbalance (Wang et al., 2018a, b), nutrient deficiency (Durbak et al., 2014), and mutations of genes including *TUTOU1,OsALMT7*, *SPL6*, *OsCIPK31*, and *DPS1* (Bai et al., 2015; Heng et al., 2018; Peng et al., 2018; Wang et al., 2018a, b; Zafar et al., 2019). When panicle apical portion in *tut1*, *spl6*, and *paa1019* mutants occurred before the heading stage, it occurred in late panicle development in the *paab1–1* and *dps1* mutants*,* similar to *paa3* in this study. With the exception of the abortion phenotype, similar to *tut1*, the seed size of the *paa3* also became smaller, while it did not change in the *paab1–1*, *spl6, paa1019*, and *dps1* mutants. In addition, a series of agronomy traits, such as panicle length, number of spikelets per panicle, setting percentage, and 1000-grain weight were all decreased in these mutants. Therefore, these studies suggested that the genes related to panicle apical portion might have a wide effect on panicle development.

Rice grains are enclosed by the hull (lemma and palea), the development of which can largely determine the final size of rice grains and then affect the rice yield and quality (Li et al., 2018; Li et al., 2019). In recent years, many studies have shown that rice size are determined by numerous genes, most of which are involving in regulation of hull development (cell differentiation and expansion) (Li et al., 2018; Li et al., 2019; Fan & Li, 2019). In our study, we observed the hull (lemma and palea) of *paa3* mutant and WT with scanning electron microscopy, and it was obvious that the hull cells of the aborted spikelet in *paa3* mutant were sharply smaller than those of WT (Fig. 2d). Then, we speculate that the hull cell extension of most of spikelets (including the fertile ones) in the *paa3* mutant might be affected, causing the small size seeds. However, it was unclear whether the *PAA3* gene regulated directly the hull development. It was possible that reduced size of grain might been indirect effect related to smaller panicle and reduced plant height in *paa3* mutant.

PCD induced by ROS accumulation in the panicle might play a key role in most panicle abortion mutants in rice. *OsALMT7* encoded an aluminum-activated malate transporter in rice. Malate was a central metabolite in the plant cell and was involved in the mitochondrial tricarboxylic acid and glyoxylate cycles in plant species, and can participate in redox reactions to produce NAD (H) or NADP (H) to maintain the balance of intracellular redox. In the *paab1–1* mutant, the loss-of-function mutant of *OsALMT7*, reduced malate might disrupted the redox balance in the panicle cells, leading to the accumulation of ROS and the death of panicle cells (Heng et al., 2018). *DPS1* encoded a CBSDUF protein, and could interact with Trx proteins (Trx1 and Trx20) to regulate ROS homeostasis in rice panicle development. Loss-of-function *DPS1* gene accumulated more ROS in *dps1* mutant defective panicles, and also induced cell death and panicle apical degeneration (Zafar et al., 2019). In this study, our results also strongly supported the involvement of *PAA3* in ROS removal, and overaccumulation of ROS triggers PCD in the apical portion of the *paa3* panicle.

*PAA3* encoded OSA7, a plasma membrane H^+^-ATPase that was a member of the ATPase superfamily. The structure of the plasma membrane H^+^-ATPase was highly conserved from fungi to higher plants, with the exception of the C-terminal region (Wang et al., 2014). Depending on the structure of the C-terminal region, the plasma membrane H^+^-ATPase could be divided into two types: the penultimate threonine (Thr)-containing H^+^-ATPase (pT H^+^-ATPase) and the non-penultimate Thr-containing H^+^-ATPase (non-pT H^+^-ATPase) (Okumura et al., 2012); OSA7 belonged to the former group. A *Tos17* insertion mutant of OSA7 resulted in impairment of the BL-induced stomatal opening, and ultimately a reduced transpiration rate, suggesting that OSA7 was involved in the Blue light-induced stomatal opening of dumbbell-shaped guard cells in monocotyledon species (Toda et al., 2016). The H^+^-ATPase is present in the membrane systems of fungi and plants widespread. In plants H^+^-ATPase releases protons from inside to outside the cell, and enhances membrane potential and pH gradient, contributing to cell wall acidification.The plasma membrane H^+^-ATPases were involved in many aspects of biology including BL-induced stomatal opening of dumbbell-shaped guard cells in the monocotyledon species, the uptake of phosphorus by the roots, and sustained pollen tube growth and fertilization (Chang et al., 2008; Toda et al., 2016; Hoffmann et al., 2020). There are 11 H^+^-ATPase subtypes in *Arabidopsis thaliana*, including AHA1 which plays an important role in the blue-light dependent stomatal opening, AHA4 which plays a role in endodermal transport of solutes, and AHA10 which is associated with seed development (Yamauchi et al., 2016; Vitart et al., 2010; Appelhagen et al., 2015). Similarly, there are 10 H^+^-ATPase in rice, including *OSHA1* gene associated with the ability to absorb nutrients, *OSHA2* may be related to photosensitive pigments and *OSHA3* which can regulate spore development. (Wang et al., 2014; Luis et al., 2020; Zhang et al., 2019). However, to date only a few H^+^-ATPase isoforms had been identified and their physiological roles have proven difficult to analyze, given that no phenotypes of H^+^-ATPase mutant have been reported. In this study, the *PAA3* mutation resulted in excessive accumulation of ROS followed by PCD in the panicle, providing a novel perspective to explore the function of H^+^-ATPases in the future. The further study of *PAA3* gene was helpful to further understand the mechanism of panicle apical abortion at the cellular level.

## Conclusions

A novel *paa3* mutant was identified in rice and showed severe panicle apical apportion and semi-dwarf. The *PAA3* encoded a H^+^-ATPase, which was a membrane protein and highly expressed in stems and panicle. The TUNEL assay showed that the DNA fragmentation and cell death in *paa3* spikelets started to occur between the stages of ~ 7 and ~ 11 cm panicles. DAB staining and measurement of H_2_O_2_ and MDA content showed overaccumulation of ROS in the *paa3* spikelets at the ~ 11 cm stage. Staining with trypan blue showed cell death in *paa3* spikelets at the stage of ~ 13 cm. The expression of genes involving in H_2_O_2_-induced PCD also indicated over-accumulation of ROS in the *paa3* spikelets. Taken together, the results indicated that *PAA3* played an important role in maintaining the panicle development through ROS removal.

## Materials and Methods

### Plant Materials and Growth Conditions

In this study, the *paa3* was derived from the ethyl methane sulfonate mutant library of the maintainer *XIDA1B* (*1B*). The *paa3* mutant was crossed with the sterile line 56 s to obtain the F_1_ generation, and the F_1_ generation was self-crossed to obtain the F2 population. All plant materials were grown in the experimental fields of the Rice Research Institute of Southwest University (Chongqing, China). All *N. benthamiana* plants were grown in a greenhouse at the Rice Research Institute of Southwest University, Chongqing, China.

### Agronomic Trait Analysis

For agronomic trait measurement, rice plants were grown in experimental fields in Chongqing, China under natural conditions. Agronomic traits (comprising internodes length, plant height, panicle length, number of primary branch, number of secondary branch, number of spikelets per panicle, number of seeds per panicle, and 1000-grain weight) for each of the *paa3* mutant and the wild type were analyzed at the mature stage with 10 replicates.

### Scanning Electron Microscopy

The panicles in both the WT and the *paa3* mutant were examined using a scanning electron microscope (SU3500; Hitachi, Tokyo, Japan) with a − 20 °C cooling stage under a low-vacuum environment, when the *paa3* panicle began to show a panicle abortion phenotype. At the flowering stage, spikelets from *paa3* and WT plants were observed using a stereomicroscope (SMZ1500; Nikon, Tokyo, Japan).

### TUNEL Assay

Apical spikelets of the WT and *paa3* panicles at the panicle lengths 7, 11, and 15 cm were collected and fixed in formalin-acetone-alcohol solution for 48 h, soaked in paraffin, embedded, sliced, and baked for 3 days. The spikelets were then dewaxed with xylene, dried, and incubated with protease K for 10 min, soaked in phosphate-buffered saline (PBS) for 5 min, and soaked in 4% methanol-free formaldehyde solution for 5 min. After adding 50 μL rTdT, the specimen was incubated at 37 °C for 3 h, followed by incubation with 2x SSC solution in the dark for 15 min and with PBS for 5 min; this procedure was repeated three times. Specimens were incubated with propidium iodide (PI) solution for 15 min to prevent infiltration, and soaked in water for 5 min; this procedure was repeated three times. Finally, the tablets were sealed with sealant (PBS and glycerin 1:1) for observation. The green fluorescence of fluorescein (TUNEL signal) and red fluorescence of propidium iodide were analysed at 488 nm (excitation) and 520 nm (detection), and 488 nm (excitation) and 610 nm (detection), respectively, under a confocal laser scanning microscope (LSM710; Zeiss, Jena, Germany).

### Staining and Quantitative Measurement of ROS and Measurement of MDA Content

We monitored cellular ROS levels in apical degenerated spikelets using DAB staining to detect H_2_O_2_. According to a method described previously (Wu et al., 2017), we used DAB to stain the top spikelets of WT and *paa3*, and quantified ROS by measuring H_2_O_2_. Fresh panicles (1 g) were collected and then measured using the H_2_O_2_ assay kit (Nanjing Jiancheng Bioengineering Institute, Nanjing, China). To measure MDA content, we collected 1 g spikelets from the apical part of the WT and *paa3* panicles. Then, according to the instructions of the MDA Assay Kit (Nanjing Jiancheng Bioengineering Institute), we measured the MDA content. All experiments were conducted on panicles at the booting stage.

### Trypan Blue Staining

Trypan blue staining was used to examine PCD. The panicles of mutant and WT at the 13 cm stage were immersed in a boiling solution of trypan blue for 10 min, and then removed and left at room temperature for 12 h. This was then immersed in a solution of chloral hydrate at a concentration of 2.5 mg/mL to allow decolourization. Finally, the decolourized samples were stored at 50% glycerin. Staining was observed using a stereomicroscope.

### Map-Based Cloning

The *paa3* mutant was crossed with the sterile line “56S” to generate F_1_. The 58 F_2_ plants that exhibited a mutant phenotype were selected as the mapping population. SSR repeat markers from publicly available rice databases, including Gramene (http://www.gramene.org), the Rice Genomic Research Program (http://rgp.dna.affrc. go.jp/public data/caps/index.html), and in/del markers designed by our group according to re-sequence of XD1B and 56S genome, were used for fine-mapping of *PAA3*. The primer sequences used for mapping and identification of transgenic plants are listed in Table S2.

### Vector Construction and Transformation

To construct the complementation plasmid, a 10,725 bp genomic fragment that contained the *PAA3* coding sequence, coupled with the 3251 bp upstream and 1068 bp downstream sequences, was amplified using the primers *PAA3*-com-F (EcoR1) and *PAA3*-com-R (HindIII). The fragment was inserted into the binary vector pCAMBIA1301 using the pEASY®-Uni Seamless Cloning and Assembly Kit (TransGen, Beijing, China). The recombinant plasmids were transformed into the *paa3* mutant using the *Agrobacterium tumefaciens*-mediated transformation method as described previously (Zhuang et al., 2020). For PAA3::GUS assays, the promoter of *PAA3* gene (3138 bp) was amplified. The fragment was inserted into the binary vector pCAMBIA1301. Then the recombinant plasmids were transformed into the japonica cultivar *ZHONGHUA 11* (*ZH11*) using the *A. tumefaciens*-mediated transformation method as described previously (Zhuang et al., 2020). The primers used for vector construction are listed in Table S2.

### *PAA3*P::GUS Staining

For promoter activity analysis, a 3138 bp genomic fragment, which is the promoter of the *PAA3* gene, was PCR-amplified from WT genomic DNA with the primer pair PAA3P-GUS-F and *PAA3*P-GUS-R (Table S2), and fused to the *GUS* reporter gene in the vector pCAMBIA1301. GUS staining was performed on PAA3P::GUS T_0_ generation transgenic plants in accordance with a previous method (Jefferson, 1989). After bleaching with ethanol, photographs were taken using a stereomicroscope.

### Subcellular Localization of the PAA3 (Transient Expression Assays in Rice Protoplasts)

The full-length coding region (without the termination codon) of *PAA3* was amplified using the SL-PAA3-F (Spe1) and SL-PAA3-R (Sma1) primers. The fragment was cloned into the expression cassette pAN580-35S:: GFP to generate the pAN580-35S::PAA3-GFP fusion vector. The pAN580-35S::GFP and pAN580-35S:: PAA3-GFP plasmids were then transformed into rice protoplasts. After incubation for 12–16 h at 28 °C, GFP fluorescence was detected using a confocal laser scanning microscope (LSM710; Zeiss, Jena, Germany). Primers used for subcellular localization are listed in Table S2.

### Subcellular Localization of the PAA3(Transient Expression in *Nicotiana benthamiana* Leaves)

The full-length coding region (without the termination codon) of *PAA3* was amplified using the YC-PAA3-F (BamH1) and YC-PAA3-R (Xba1) primers. The fragment was cloned into the expression cassette pCAMBIA1300-35S::eGFP to generate the pCAMBIA-35S::PAA3-eGFP fusion vector. The pCAMBIA1300-35S::eGFP and pCAMBIA-35S::PAA3-eGFP plasmids were then transformed into *Agrobacterium tumefaciens* strain GV3101. And then *Agrobacterium tumefaciens* strain GV3101containing the target plasmid was grown in YEB medium with antibiotic selection to OD_600_ = 0.6–0.8. Cells were suspended to an OD_600_ = 0.4 in MES buffer (10 mM MgCl_2_, 10 mM MES; pH 5.6) and kept in the dark for 2–4 h before inoculation. Leaves were analyzed at 2–3 days after transformation. Primers used for subcellular localization are listed in Table S2.

### DNA Extraction, RNA Isolation, and qPCR

Total DNA from WT and *paa3* mutant was extracted using the CTAB method. Total RNA from root, stem, leaf, sheath, panicle, bud, and shoot was isolated using the RNA prep Pure Plant Kit (Tiangen, Beijing, China). The first-strand complementary cDNA was synthesized from 2 μg total RNA using oligo (dT)_18_ primers in a 20 μL reaction volume using the PrimeScript® Reagent Kit with gDNA Eraser (Takara, Dalian, China). The qPCR analysis was performed using the SYBR® Premix Ex Taq™ II Kit (Takara) in the ABI 7500 Sequence Detection System (Applied Biosystems, Carlsbad, CA, USA). *ACTIN* (*OsRac1*, *LOC_Os01g12900*) was used as the endogenous control. At least three replicates were performed. Primers used for qPCR are listed in Table S2.

### RNA-Sequencing Analysis

Analysis of RNA sequencing (RNA-seq) data was performed using a standard protocol (Trapnell et al., 2012). For RNA-seq analysis, RNA was extracted from WT and *paa3* panicles of 11 cm in length that corresponded to the developmental stage just after the start of panicle abortion. All sequencing samples were treated. RNA sequencing was performed by Novogene Biotechnology (Beijng, China), and sequencing data were retrieved through the standard Illumina pipeline with custom and default parameters. HTSeq software was used to analyse the original sequences of known genes for all the samples (Novogene Biotechnology, Beijing, China), and the expression of known genes was calculated using the fragments per kilobase of transcript per million fragments mapped (FPKM). HTSeq was used to estimate gene expression levels. DEGs were identified considered *P* ≤ 0.05 and a log2 fold-change ≥1. Clusters were analysed by principal component analysis, and DEGs were analysed by DESeq, with a cut-off P ≤ 0.05 and fold change ≥2. GO analysis and Kyoto Encyclopedia of Genes and Genomes analyses were performed to identify the significantly enriched biological processes in *paa3*.

## Supplementary Information

**Additional file 1: Fig. S1**. Identification of transgenic plants. a: Two pairs of primers for amplification to detect the 15 transformants (comF1-GUSR1 for exogenous vector; comF2-comR2 for endogenic sites). b, Comparison diagram of sequence.

**Additional file 2: Fig. S2**. Transient expression in *Nicotiana benthamiana* leaves. Upper row indicates the expression of GFP protein without PAA3 in N *benthamiana* leaves as the negative control. Lower row indicates the plasma membrane localization of the PAA3 protein in N *benthamiana* leaves given by the expression of PAA3 fused with GFP. Green is GFP signal. The plasma membrane as labelled by MF4–64. Red is the chloroplast signal. Bars: 20 μm.

**Additional file 3: Fig. S3**. Trypan blue staining analysis. a, Top panicle of *paa3* mutants and WT. (panicle length = 13 cm). b, Top spikelet of *paa3* mutants and WT. Bars: (a, b) 5 mm.

**Additional file 4: Fig. S4**. DAB staining analysis. a, Top panicle of *paa3* mutants and WT. (panicle length = 13 cm). b, Top spikelet of *paa3* mutants and WT. Bars: (a, b) 5 mm.

**Additional file 5: Fig. S5**. Transcriptome analysis of *paa3* mutant. a: It showed that there were 1075 differential genes in WT and *paa3* samples, among which 991 genes were up-regulated and 84 genes were down-regulated. b: The volcano figure showed an overall overview, including 28,317 genes with no change in expression, 991 up-regulated genes and 84 down-regulated genes.

**Additional file 6: Table S1**. Total frequency of spikelet abortion of *paa3* mutant.

**Additional file 7: Table S2**. All primers used for this study.

## Data Availability

The datasets supporting the conclusions of this article are included within the article and its additional files.

## Abbreviations

*PAA3*: *PANICLE APICAL ABORTION 3*
WT: Wild-type
*paa3*: *Panicle apical abortion 3*
AMs: Axillary meristems
PB: Primary branches
SB: Secondary branches
LS: Lateral spikelet
TS: Terminal spikelet
QTL: Quantitative trait locus
ROS: Reactive oxygen species
PCD: Programmed cell death
TUNEL: Terminal deoxynucleotidyl transferase-mediated dUTP nick-end labeling
BSA: Bulked segregant analysis
MDA: Malondialdehyde
SSR: Simple sequence repeat
DAB: 3,3′-Diaminobenzidine
EMS: Ethyl methane sulfonate
CTAB: cetyltrimethylammonium bromide
GO: Gene ontology
KEGG: Kyoto encyclopedia of genes and genomes
GFP: Green fluorescent protein
GUS: β- Glucuronidase
qPCR: Quantitative PCR
TEM: Transmission electron microscopy
