# Supplementary material for: Panicle Apical Abortion 3 Controls Panicle Development and Seed Size in Rice
Source: Rice (N Y). 2021 Jul 15;14:68. doi: 10.1186/s12284-021-00509-5 (PMC8282854; doi:10.1186/s12284-021-00509-5)
Supplement: Supplementary file 1 — Additional file 1: Fig. S1. Identification of transgenic plants. a: Two pairs of primers for amplification to detect the 15 transformants (comF1-GUSR1 for exogenous vector; comF2-comR2 for endogenic sites). b, Comparison diagram of sequence. [file 12284_2021_509_MOESM1_ESM.pdf]

Supplemental Figure 1

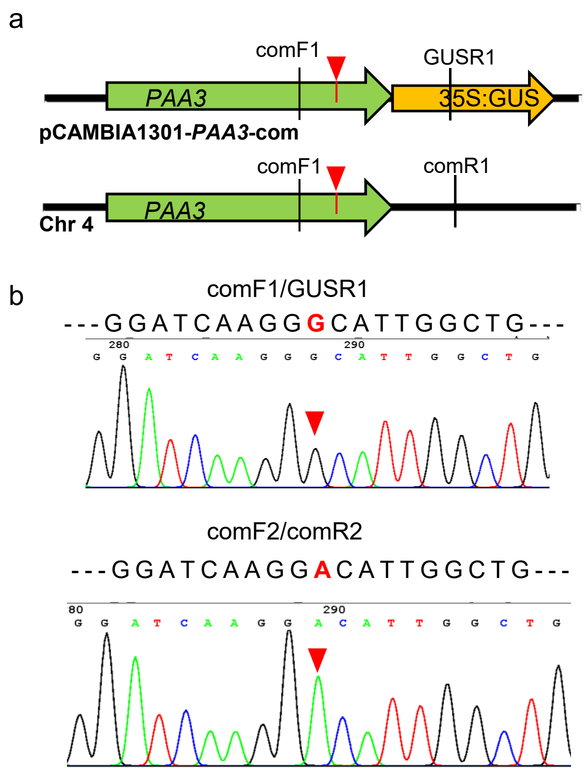

Fig. S1, Identification of transgenic plants. a, Two pairs of primers for amplification to detect the 15 transformants (comF1-GUSR1 for exogenous vector; comF2-comR2 for endogenic sites).b, Comparison diagram of sequence.
