# Supplementary material for: Panicle Apical Abortion 3 Controls Panicle Development and Seed Size in Rice
Source: Rice (N Y). 2021 Jul 15;14:68. doi: 10.1186/s12284-021-00509-5 (PMC8282854; doi:10.1186/s12284-021-00509-5)
Supplement: Supplementary file 2 — Additional file 2: Fig. S2. Transient expression in Nicotiana benthamiana leaves. Upper row indicates the expression of GFP protein without PAA3 in N benthamiana leaves as the negative control. Lower row indicates the plasma membrane localization of the PAA3 protein in N benthamiana leaves given by the expression of PAA3 fused with GFP. Green is GFP signal. The plasma membrane as labelled by MF4–64. Red is the chloroplast signal. Bars: 20 μm. [file 12284_2021_509_MOESM2_ESM.pdf]

Supplemental Figure 2

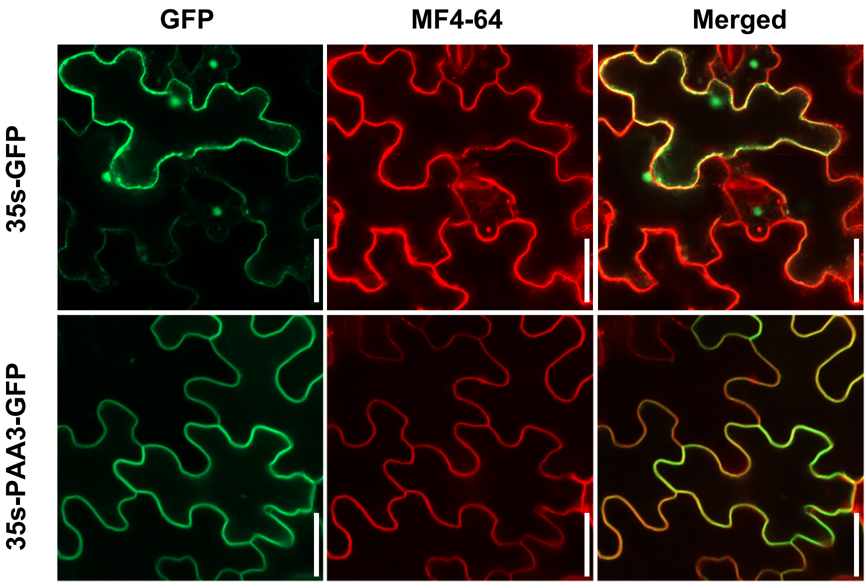

Fig. S2 Transient expression in *Nicotiana benthamiana* leaves. Upper row indicates the expression of GFP protein without PAA3 in N *benthamiana* leaves as the negative control. Lower row indicates the plasma membrane localization of the PAA3 protein in N *benthamiana* leaves given by the expression of PAA3 fused with GFP. Green is GFP signal. The plasma membrane as labelled by MF4-64. Red is the chloroplast signal. Bars: 20μm
