# Supplementary material for: Panicle Apical Abortion 3 Controls Panicle Development and Seed Size in Rice
Source: Rice (N Y). 2021 Jul 15;14:68. doi: 10.1186/s12284-021-00509-5 (PMC8282854; doi:10.1186/s12284-021-00509-5)
Supplement: Supplementary file 4 — Additional file 4: Fig. S4. DAB staining analysis. a, Top panicle of paa3 mutants and WT. (panicle length = 13 cm). b, Top spikelet of paa3 mutants and WT. Bars: (a, b) 5 mm. [file 12284_2021_509_MOESM4_ESM.pdf]

Supplemental Figure 4

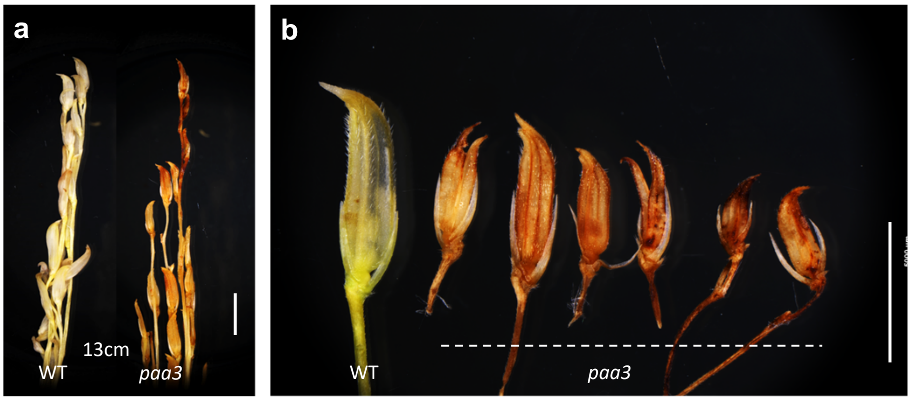

Fig. S4 DAB staining analysis. a, Top panicle of *paa3* mutants and WT. (panicle length=13 cm). b, Top spikelet of *paa3* mutants and WT. Bars: (a, b) 5mm
