# Supplementary material for: Panicle Apical Abortion 3 Controls Panicle Development and Seed Size in Rice
Source: Rice (N Y). 2021 Jul 15;14:68. doi: 10.1186/s12284-021-00509-5 (PMC8282854; doi:10.1186/s12284-021-00509-5)
Supplement: Supplementary file 5 — Additional file 5: Fig. S5. Transcriptome analysis of paa3 mutant. a: It showed that there were 1075 differential genes in WT and paa3 samples, among which 991 genes were up-regulated and 84 genes were down-regulated. b: The volcano figure showed an overall overview, including 28,317 genes with no change in expression, 991 up-regulated genes and 84 down-regulated genes. [file 12284_2021_509_MOESM5_ESM.pdf]

Supplemental Figure 5

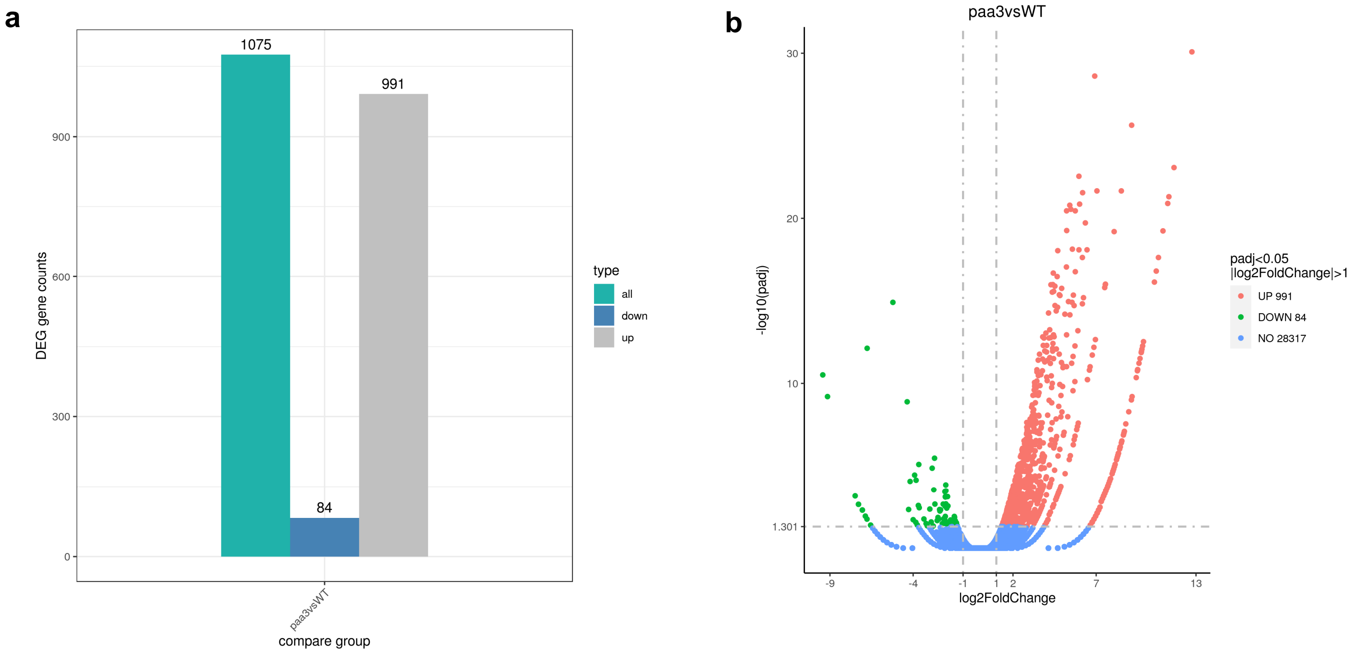

Fig. S5, **transcriptome analysis of *paa3***. a, Showed that ther e were 1075 differential genes in WT and *paa3* samples, among which 991 genes were up-regulated and 84 genes were down-regulated. **b** The volcano figure showed an overall overview, including 28,317 genes with no change in expression, 991 up-regulated genes and 84 down-regulated genes.
