# Supplementary material for: Panicle Apical Abortion 3 Controls Panicle Development and Seed Size in Rice
Source: Rice (N Y). 2021 Jul 15;14:68. doi: 10.1186/s12284-021-00509-5 (PMC8282854; doi:10.1186/s12284-021-00509-5)
Supplement: Supplementary file 6 — Additional file 6: Table S1. Total frequency of spikelet abortion of paa3 mutant. [file 12284_2021_509_MOESM6_ESM.pdf]

Supplemental Table 1 Total frequency of spikelet abortion of *paa3* mutant											
**Sample**	**Number of spikelets per panicle**	**Number of seeds per panicle**	**Abortion rate**	**Average abortion rate**	**SD**	**Sample**	**Number of spikelets per panicle**	**Number of seeds per panicle**	**Abortion rate**	**Average abortion rate**	**SD**
**1B-1**	206	204	0.97%	1.25%	1.00%	***paa3* -1**	213	172	19.25%	18.25%	4.97%
**1B-2**	229	225	1.75%			***paa3* -2**	203	176	13.30%		
**1B-3**	211	209	0.95%			***paa3* -3**	202	164	18.81%		
**1B-4**	205	203	0.98%			***paa3* -4**	230	183	20.43%		
**1B-5**	197	194	1.52%			***paa3* -5**	242	165	31.82%		
**1B-6**	200	199	0.50%			***paa3* -6**	156	130	16.67%		
**1B-7**	210	210	0.00%			***paa3* -7**	226	191	15.49%		
**1B-8**	209	201	3.83%			***paa3* -8**	238	201	15.55%		
**1B-9**	196	193	1.53%			***paa3* -9**	226	190	15.93%		
**1B-10**	229	228	0.44%			***paa3* -10**	184	156	15.22%		
