# Supplementary material for: Panicle Apical Abortion 3 Controls Panicle Development and Seed Size in Rice
Source: Rice (N Y). 2021 Jul 15;14:68. doi: 10.1186/s12284-021-00509-5 (PMC8282854; doi:10.1186/s12284-021-00509-5)
Supplement: Supplementary file 7 — Additional file 7: Table S2. All primers used for this study. [file 12284_2021_509_MOESM7_ESM.pdf]

Supplemental Table 2 All primers for this study

Primer Name	Sequence(5'-3')	Purpose
ZTQ38F	TGCGAATGCTTGAATGGATC	Fine mapping of the *PAA3* gene
ZTQ38R	CAAGGCTCACTTCCTAGATCAAG	
CHR4-P118-3F	GGTAGCATCCATTTGCCGA	
CHR4-P118-3R	GTGACGGCTCGAACATCTG	
CHR4-P118-6F	CGGATTGAACTGTCCATGTTAAC	
CHR4-P118-6R	CCTTCACAATGATGAATGGTC	
CHR4-P118-9F	GATATGATATGATCGAGCCGCTCG	
CHR4-P118-9R	CTGATGCAGCTTACATTGCTGG	
PAA3 -com-F(EcoR1)	ATGACCATGATTACGAATTCTGAGG TCATCCAATCAGACGCTGAC	Complementary test
PAA3 -com-R(HindIII)	CGACGGCCAGTGCCAAGCTTCCATG CCGAGTGCAATGCTACT	
comF1	CACTATTACAATTCTAGCAACGACC	Identification of transgenic plants
GUSR1	CTGCCTGGCACAGCAATTG	
comF2	CAAATTCGGTGTCAGATCAATC	
comR2	GCCAGCGTACTGTATTCGCCTC	
PAA3 P-GUS-F(*EcoR1*)	ATGACCATGATTACGAATTCTGAGG TCATCCAATCAGACGCTGAC	Expression pattern analysis
PAA3 P-GUS-R(*Nco1*)	TACCCTCAGATCTACCATGGCGGTG GTGGTATTAGTAGTGGAGAGG	
SL- *PAA3* -F（*Spe1* ）	GACAGCCCAGATCAACTAGTATGGG TGGGCTCGAGGAGAT	Subcellular localization
SL- *PAA3* -R (*Sma1*)	CTCACCATGGATCCCCCGGGCACTG TGTAGTTCTGCTGGATCG	
YC- PAA3-F（*BamH1* ）	GAGCTCGGTACCCGGGGATCCATGG GTGGGCTCGAGGAGATCA	
YC- PAA3-R (Xba1)	TTGCTCACCATGTCGACTCTAGACA CTGTGTAGTTCTGCTGGAT	
q-*PAA3* F	CAAGGACGCTTCACTTGAGGC	For qRT-PCR
q-*PAA3* R	GCAATACCAATGTCGGCCTTC	
q-*OsVPE2* F	CGGCTCCAACGGCTACTACAAC	
q-OsVPE2R	TCGGGACCCCAGCATAGACA	
q-*OsVPE3* F	CGGTAACTACAGGCACCAGGC	
q-*OsVPE3* R	GTGACTTCGTCTCCAGTGTAATCC	
q-*CATA* F	CCACCGATTACCGCCACA	
q-*CATA* R	GCCTCGTCGTCCATCAAGC	
q-*CATB* F	ATCCCACGCAACTACAGG	
q-*CATB* R	CAAGGTGAGGCTTTCCAT	
q-*CATC* F	CCGCTTCTCCACCGTCAT	
q-*CATC* R	ACCAGGTCCCAGTTGCCC	
q-*ACTIN* F	TGGCATCTCTCAGCACATTCC	
q-*ACTIN* R	TGCACAATGGATGGGTCAGA	
q*LOC_Os04g10160* F	GGTTCATCGACGCCATGAC	
q*LOC_Os04g10160* R	GCCCTGATCTCTGATCCTGA	
q*LOC_Os01g43750* F	CGAAGGCGAGAAGTGGGTC	
q*LOC_Os01g43750* R	GAACCAAGGGACTCCATCCAC	
q*LOC_Os11g29290* F	CCGTGATGGGGCTCATCC	
q*LOC_Os11g29290* R	GTACCCGCATTCGATCATC	
q*LOC_Os02g36030* F	GTGCTCAACCTCGTCTCCAGC	
q*LOC_Os02g36030* R	CGACGAGGTCCTCCACGAG	
q-*LOC_Os04g59190* F	GCGCTCCAGCGTGAGATC	
q-*LOC_Os04g59190* R	CGCCACTCAAGTAAACCGA	

q-*LOC_Os04g59150* F	CTGAAGCTCATCGAGGACATC	
q-LOC_Os04g59150R	GAGGCACGTCGAAGTAGGG	
q-*LOC_Os01g73200* F	CCAGTTTCGCGTCTGAGG	
q-*LOC_Os01g73200* R	GCGTCGAGCTTGAGCTTG	
q-*LOC_Os04g59260* F	GTGCAAGCCGCTCTCCAG	
q-*LOC_Os04g59260* R	CTGCTCCGAGTTGCTGCCTC	
